# Orthodontic Aligner Incorporating *Eucommia* *ulmoides* Exerts Low Continuous Force: In Vitro Study

**DOI:** 10.3390/ma13184085

**Published:** 2020-09-14

**Authors:** Sayuri Inoue, Satoshi Yamaguchi, Hiroshi Uyama, Takashi Yamashiro, Satoshi Imazato

**Affiliations:** 1 Department of Biomaterials Science, Osaka University Graduate School of Dentistry, 1-8 Yamadaoka, Suita, Osaka 565-0871, Japan; sayuri-inoue@dent.osaka-u.ac.jp (S.I.); imazato@dent.osaka-u.ac.jp (S.I.); 2 Department of Orthodontics and Dentofacial Orthopedics, Osaka University Graduate School of Dentistry, 1-8 Yamadaoka, Suita, Osaka 565-0871, Japan; yamashiro@dent.osaka-u.ac.jp; 3 Division of Applied Chemistry, Osaka University Graduate School of Engineering, 2-1 Yamadaoka, Suita, Osaka 565-0871, Japan; uyama@chem.eng.osaka-u.ac.jp

**Keywords:** orthodontic appliance, thermoplastics, *Eucommia ulmoides*, orthodontic wires

## Abstract

The aim of this study was to investigate the orthodontic force exerted by thermoplastic orthodontic appliances incorporating *Eucommia* *ulmoides* in terms of usefulness as the aligner-type orthodontic device. Erkodur, Essix C+^®^, *Eucommia* elastomer, and edgewise brackets were used (*n* = 3, each; thickness = 1.0 mm, each). The orthodontic force on the upper right incisor was measured every 24 h for two weeks using a custom-made measuring device. The force of the *Eucommia* elastomer (4.25 ± 0.274 N) and multi bracket system (5.32 ± 0.338 N) did not change from the beginning to the end (*p* > 0.01). The orthodontic force exerted by the *Eucommia* elastomer was lower than that of the multi-bracket orthodontic appliance from the beginning to the end. The force of Erkodur significantly decreased from the beginning to 24 h (6.47 ± 1.40 N) and 48 h (3.30 ± 0.536 N) (*p* < 0.01). The force of Essix C+^®^ significantly decreased from the beginning (13.2 ± 0.845 N) to 24 h (8.77 ± 0.231 N) (*p* < 0.01). The thermoplastic orthodontic appliance made of *Eucommia* elastomer continuously exerted a constant orthodontic force for two weeks under water immersion conditions. The orthodontic force of *Eucommia* elastomer was found to be similar to the orthodontic force exerted by the multi-bracket orthodontic appliance with 0.019 × 0.025 in nickel–titanium wire. These results suggest that the *Eucommia* elastomer has possibly become one of the more useful materials to form thermoplastic orthodontic appliance exerting low continues orthodontic force.

## 1. Introduction

Orthodontic treatment using a transparent thermoplastic orthodontic appliance, called aligner-type orthodontic devices, has recently increased with the aesthetic demands of patients. Aligner-type orthodontic devices appeared in the early 1970s as a retainer for preventing relapse of the teeth after orthodontic treatment [1], then they started to be used for minor tooth movement [2]. To date, it has been devised in a variety of materials and thicknesses for efficient orthodontic treatment [3,4,5]. At present, the method for fabricating an aligner using a digital setup model designed by simulating tooth movement via a computer has become widespread, and orthodontic treatment by aligner has expanded the range of application from minor tooth movement to whole teeth orthodontic treatment to full jaw treatment [6,7,8,9]. With the spread of orthodontic treatment using thermoplastic orthodontic appliances, many experiments have been conducted to investigate the orthodontic force exerted by such appliances. At present, the most thermoplastic materials used for aligners are made by polyethylene terephthalate glycol (PETG), but there are other materials made of polyurethane (PU) or polyethylene terephthalate (PET) [4,10]. Furthermore, new thermoplastic materials have also been developed such as by mixing PETG, PU, and polycarbonate at unique blending ratios [4] and by using PU with shape memory functions [11].

However, the amount of tooth movement using an aligner is limited to a range of 0.25 mm to 1.0 mm [3,12], so approximately 50 aligners may be required to complete orthodontic treatment. In addition, after setting an aligner on dentition, the teeth are subjected to an excessive orthodontic force, and then the force decreases over time. In the current treatment system, a new aligner must be replaced every 10 to 14 days.

Therefore, to make orthodontic treatment with an aligner more efficient, the development of a new thermoplastic materials that can continuously exert suitable orthodontic force for tooth movement are required.

*Eucommia* elastomer (EE), a trans-1,4-polyisoprene extracted and purified from *Eucommia ulmoides*, is a highly biosafety polymer exhibiting high tensile properties and large breaking strain [13]. As a tensile property, it shows high stress to initial strain, but after that it shows constant stress even if strain increases. Therefore, it can be expected that an aligner made from EE is suitable for tooth movement and be able to exert a constant orthodontic force. However, there have only been a few studies of the physical properties of the thermoplastic orthodontic materials [4,10,14,15,16,17,18] and the orthodontic force exerted by the aligner [19,20,21,22,23]. In this study, we focused on the high ductility of EE and aimed to evaluate an aligner-type orthodontic device that is suitable for the movement of teeth and that expresses weak and sustained orthodontic force.

The aim of this study was to investigate the orthodontic force exerted by thermoplastic orthodontic appliances incorporating *Eucommia ulmoides* in terms of usefulness as the aligner-type orthodontic device.

## 2. Materials and Methods 

### 2.1. Specimen Preparation

Erkodur (ER) (ERKODENT Erich Kopp GmbH, Pfalzgrafenweiler, Germany), Essix C+^®^ Plastic (EC) (DENTSPLY Raintree Essix, Sarasota, FL, USA), and (EE) (Hitachi Zosen, Osaka, Japan) were used. Trans-1,4-polyisoprene powder extracted and purified from *Eucommia ulmoides* was fused and was formed as EE by mold injection. All of these materials were 1.0 mm thick single-layer materials with 120.0 mm in diameter (Table 1).

**Figure 1:**
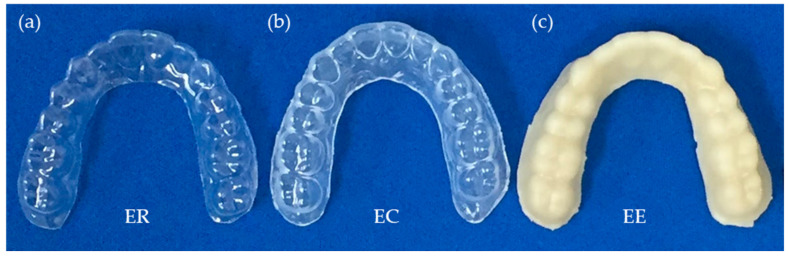
(**a**) Aligner formed using ER; (**b**) aligner formed using EC, and (**c**) aligner formed using EE.

The standard dental model (E50-500AU, NISSHIN, Kyoto, Japan) was duplicated using a 3D scanner (S-WAVE D900, SHOFU, Kyoto, Japan) and 3D printer (AGILISTA-3200, KEYENCE, Osaka, Japan) as the normal dentition. The aligners were formed using ER and EC according to the instructions of the manufacturer (*n* = 3, each). The ER was heated to 160 °C, pressed to the model, and cooled for 45 s using a vacuum-forming machine (Elkopress 300Tp, ERKODENT Erich Kopp GmbH, Germany) (Figure 1). The EC was heated to 220 °C for 50 s, pressed to the model and cooled for 120 s using a vacuum-forming machine (Biostar VII JM Ortho, Tokyo, Japan) (Figure 1). The EE was heated to 220 °C for 20 s, pressed to the model, and cooled for 240 s using the same vacuum-forming machine as EC (Figure 1).

### 2.2. Characterization of Eucommia Elastomer

Five round specimens (ϕ15 × 1.0 mm) of EE was prepared and water absorption test was conducted according to ISO 4049:2019 [24] (*n* = 5). Test specimens were prepared similar to the water absorption test and subjected to X-ray diffraction (XRD) analysis (Rint2000, Rigaku, Tokyo, Japan) in dry condition The XRD analysis was conducted at 2θ range between 2° and 60° with a step size of 0.02° in a continuous mode of 4.0°/min. Fourier transformation infrared spectroscopy (FTIR) was conducted. Part of each material (6.0 × 6.0 × 1.0 mm) was prepared and the spectra were recorded with an FTIR spectrometer (FT-IR 8300, Shimadzu, Kyoto, Japan) and averaged over 20 scans between 700–4000 cm^−1^. Ten specimens of 1.0 mm thickness were prepared according to ISO 527-2 type 5B [25]. Five specimens were immersed in distilled water at 37 °C with no strain for 24 h and two weeks. The remaining specimens were loaded to 1% strain with the custom-made loading device developed in previous study [18] and were stored in distilled water at 37 °C for two weeks. Tensile tests were conducted with a universal testing machine (EZ-SX, Shimadzu, Kyoto, Japan) with a 500 N load cell, and an elastic modulus was determined from the slope of the obtained stress–strain curve. Physical properties of ER and EC have been measured in a previous study [18] and compared to those of EE.

### 2.3. Evaluation of Orthodontic Force 

**Figure 2:**
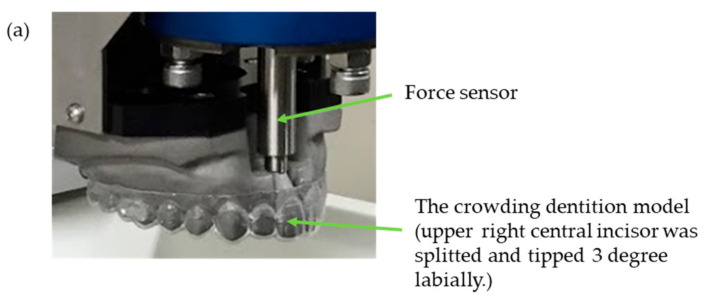

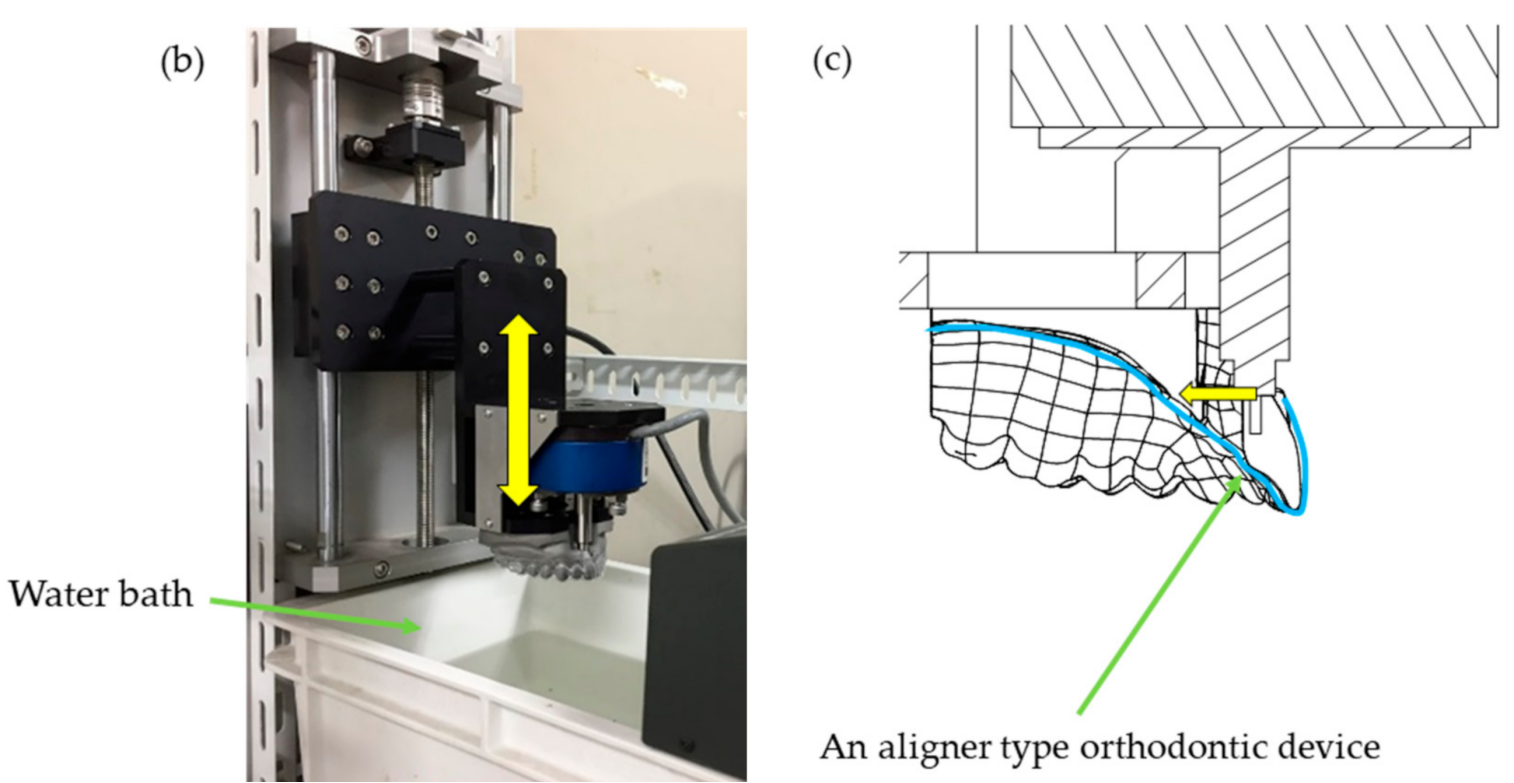
Custom-made device for measuring orthodontic force. (**a**) The upper right central incisor was tilted 3° to labially, a force sensor was installed at the root 1/2 position, and an ER or EC aligner was put on the model. (**b**) The model was fixed to a linear actuator and immersed in a constant temperature bath at 37 °C by adjusting the height (yellow arrow in the figure). (**c**) The model and force sensor. The orthodontic force in the direction of the yellow arrow expressed by the aligner-type orthodontic device (light blue line) was measured at a cycle of 10 times/s.

Using 3D data of the standard model (E50-500AU, Nisshin, Kyoto, Japan), the stainless-steel-made dentition model was prepared where the upper right central incisor was tipped 3° labially (TRUESEED, Kyoto, Japan) as the crowded dentition. A force sensor (Nitta, Osaka, Japan) was attached to at the middle of root of the upper right central incisor (Figure 2a). Aligner-type orthodontic devices using thermoplastic materials were applied to the dentition model, and they were immersed to 37 °C distilled water using a custom-made linear actuator (TRUESEED) (Figure 2b,c) to replicate the oral cavity conditions. Using the actuator, the orthodontic force on upper right incisor was measured every 24 h for two weeks.

### 2.4. Multi-Bracket Orthodontic Appliance Model

**Figure 3:**
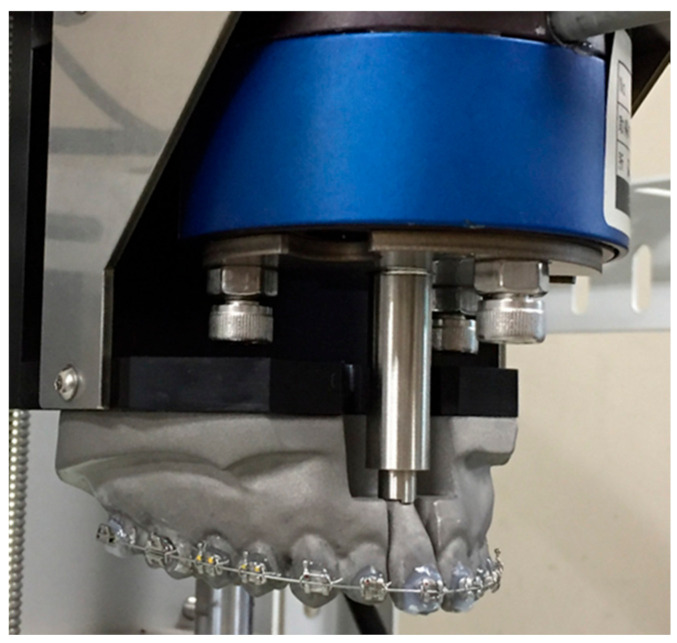
A multi-bracket orthodontic appliance model.

To compare the force, the orthodontic force exerted by multi-bracket orthodontic appliances using nickel titanium wire and metal brackets was measured using the same dentition model. The 0.022 × 0.028 inch slot brackets (VictoryTM, 3M Japan, Tokyo, Japan) were attached to the dentition model using light-cure orthodontic adhesive system (Beauty Ortho Bond Ⅱ, SHOFU, Kyoto, Japan) and metal primer (METHAL PRIMER Z, GC, Tokyo, Japan). The 0.019 × 0.025 in nickel titanium wire (Naitenol classic archwire, 3M Japan, Tokyo, Japan) were prepared and inserted to the dentition model (Figure 3). They were also immersed 37 °C distilled water for two weeks and the force were measured every 24 h.

### 2.5. Statistical Analysis

The mean amount of water absorption of EE after 24 h and two weeks were analyzed by the Student’s *t*-test. The mean elastic modulus of EE after 24 h without strain and two weeks with 1% constant strain were analyzed by the Student’s *t*-test. The *p*-values of less than 0.05 were considered as statistically significant. The mean force in each group were statistically analyzed by one-way analysis of variance (ANOVA) and Dunnett’s test (PASW Statistics 18, IBM, Somers, NY, USA). The *p*-values of less than 0.01 were considered as statistically significant.

## 3. Results

**Figure 4:**
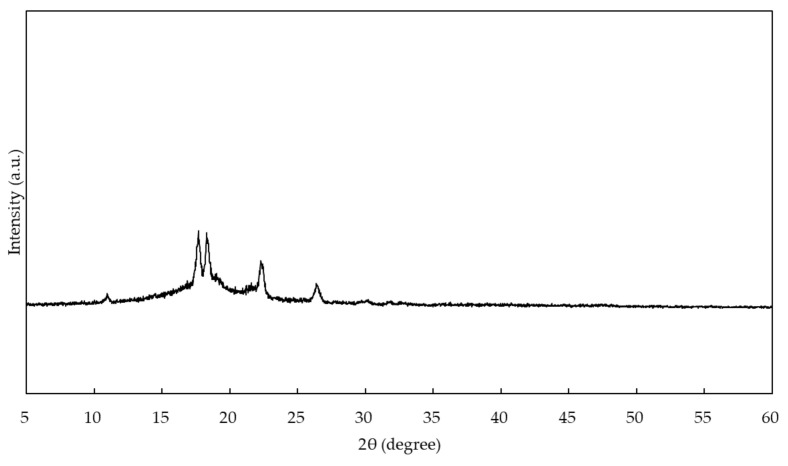
X-ray diffraction pattern of EE.

**Figure 5:**
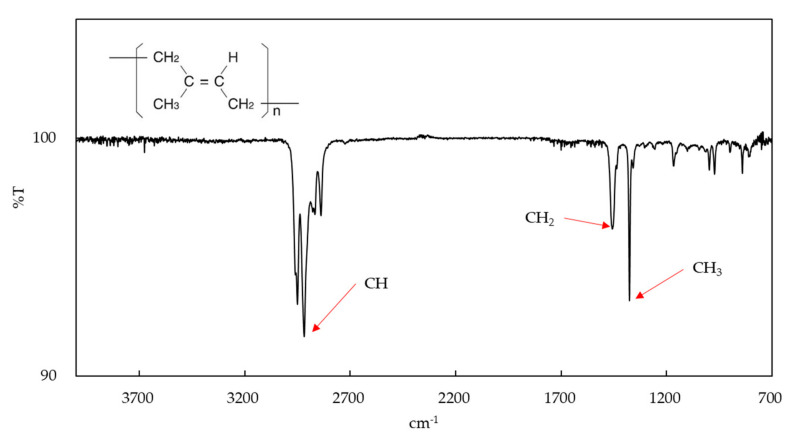
Infrared absorption spectrum for EE.

The amount of water absorption of EE after 24 h and two weeks were 0.0344 ± 0.4291 μg/mm^3^ and 0.8761 ± 0.3730 μg/mm^3^, respectively, and a significant difference was found (*p* < 0.05). The XRD patterns of EE are shown in Figure 4. Diffraction patterns indicated that EE was a crystalline polymer same as EC [17]. The infrared absorption spectrum obtained by FTIR analysis of EE showed molecular structure based on covalent bond between carbon and hydrogen (Figure 5). The elastic modulus of EE when immersed in distilled water for 24 h without strain and two weeks with 1% constant strain were 137.4 ± 6.867 MPa and 131.8 ± 11.09 MPa, respectively. There was no significant difference in both elastic moduli (*p* > 0.05).

**Figure 6:**
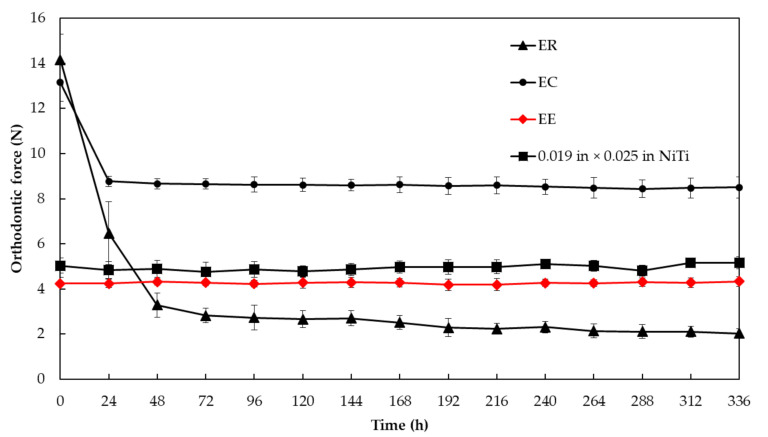
Orthodontic force measured from each material.

Figure 6 shows the force of each aligner-type orthodontic device and multi bracket system obtained by the custom-made orthodontic force measurement device. The force of EE (4.25 ± 0.274 N) and multi bracket system (5.32 ± 0.338 N) did not change from the beginning to the end (*p* > 0.01). The orthodontic force exerted by EE was lower than that of multi-bracket orthodontic appliance from the beginning to the end. The force of ER was significantly decreased from the beginning to 24 h (6.47 ± 1.40 N) and 48 h (3.30 ± 0.536 N) (*p* < 0.01). The force of EC was significantly decreased from the beginning (13.2 ± 0.845 N) to 24 h (8.77 ± 0.231 N) (*p* < 0.01).

## 4. Discussion

Various measuring devices have been reported for orthodontic force [17,19,21,22,23]. In this study, an orthodontic force measurement device was designed which can measure the reaction force generated at the middle of root of the upper right central incisor using a force sensor, and the force was measured. Our measuring device is unique because there is no previous research that measured the force while immersing in water with the aligner attached to the dental arch. This experiment simulated the same dentition model also in the multi-bracket orthodontic appliance as the thermoplastic orthodontic appliance, it was possible to directly compare it with the force exerted by EE aligner. In addition, by measuring the force for two weeks, it was possible to clarify the change of the force exerted by the thermoplastic orthodontic appliances. In ER and EC, the force in the first 24 to 48 h significantly decreased. Li et al. [19] measured the force over the time for two weeks when an aligner made of 1.0 mm thick ER was attached to a dental model twice the size in which the upper right central incisor was inclined to labial side. It was reported that a significant decrease force was observed in the first eight hours [19]. Fang et al. [14] reported that a stress relaxation test was conducted by applying 5% constant strain to a dumbbell-shaped specimen of ER, and that stress relaxation was promoted under water immersion condition. The results of our study showing that the force of ER decreased early in the period are consistent with these reports.

The EE demonstrated a lower amount of water absorption than both ER and EC [18]. Also, the EE was a crystalline polymer tending to low water absorption such as EC [18]. Even when immersed in distilled water for two weeks with constant strain, the elastic modulus of EE was not varied and lower than those of ER and EC [18]. The EE aligner used in this experiment did not change its force for two weeks after the start of the measurement and exhibited similar properties as the nickel titanium wire. These results suggest that an aligner made from EE may be able to continue tooth movement for more than two weeks, and it is expected that the replacement period can be set to two weeks or more, unlike the existing system. 

In ER, it has been reported that the force that exerts as tooth displacement increases also increases [19,21,22]. On the other hand, the EE has high ductility, so even if the displacement amount increases, the force that is developed does not increase, and it may be possible to set the movement amount of teeth with one device large.

The optimal correction force for multi-bracket devices is said to be in the range of 0.343 to 0.980 N [26] or not exceed 0.588 N [27]. The force of EE and nickel–titanium wire obtained by our research was higher than those. Barbagallo [20] measured force when the maxillary first premolar tipped buccal side by 0.5 mm using an aligner made of ER with a thickness of 0.8 mm for patients with moderate crowding using a film sheet capable of measuring the pressure [20]. The orthodontic force that was initially developed was 5.12 N, and 1.2N after two weeks of wear. These forces were also greater than the force suggested by Proffit et al. [26] and Reitan [27] above. That is, even if movement of the teeth and buffering of the force by other factors, such as the periodontal ligament and the alveolar bone occur in the body, the initial force developed by the aligner is basically larger than the nickel–titanium wire. Excessive force can contribute to root resorption that occurs during orthodontic treatment [28].

Schwarz [29] has reports that when a load of more than 0.255 N per unit area is applied to the root, an anemia is formed in the periodontal ligament and the root is absorbed. Based on Jepsen’s [30] report that the root surface area of the first premolar is 2.34 ± 0.33 cm^2^ [30], it can be calculated that root resorption occurs in the first premolar when it exceeds 0.681 N, but the value indicated by Barbagallo [20] exceeded the force. On the other hand, Gay et al. [31] and Iglesias-Linares et al. [32] reported that the frequency of root resorption in patients treated with aligner-type orthodontic devices made from PU was not significantly different from that using multi-bracket devices. A variety of factors are involved in root resorption such as patient’s gene characteristics [33], dynamic length of orthodontic treatment [34], tooth movement direction [35,36]. For this reason, it is difficult to evaluate its usefulness based on the orthodontic force alone, but it will be necessary to further study.

In addition, the thermoplastic materials’ wear, perforation, and cracking of the aligner has been reported as an issue [37,38], and the deposition of calcium phosphate [39] was induced when using an aligner. In the future, further verification experiments will be required for clinical application of EE including wear tests.

Furthermore, the EE is made from a natural plant and biocompatibility was confirmed according to ISO 10993 [40,41]; it is eco-friendly compared to conventional thermoplastic orthodontic materials. As the main limitation of this study, the EE aligner is yellowish due to the fact of its original color of the plant as shown in Figure 1c and thus improvements to make it white or transparent are necessary when considering clinical application. Moreover, the EE has a weak to high temperature and paying attention to the heat for molding was required.

## 5. Conclusions

The thermoplastic orthodontic appliances incorporating *Eucommia ulmoides* continuously exerted a constant orthodontic force for two weeks under water immersion conditions. The orthodontic force of *Eucommia* elastomer was found to be similar to the orthodontic force exerted by the multi-bracket orthodontic appliance with 0.019 × 0.025 in nickel–titanium wire. Those results suggest that the *Eucommia* elastomer is possibly become one of useful materials to form thermoplastic orthodontic appliance exerting low continues orthodontic force.

## Figures and Tables

**Table 1:** Orthodontic thermoplastic materials used in this study.

Product	Code	Manufacturer	Composition
Erkodur	ER	Erkodent Erich Kopp GmbH	Polyethylene terephthalate glycol (PETG)
Essix C+^®^ Plastic	EC	DENTSPLY Raintree Essix	Polypropylene (PP)
*Eucommia* elastomer	EE	Hitachi Zosen	*Eucommia ulmoides*
